# Evaluation of the Bactericidal Activity of Plazomicin and Comparators against Multidrug-Resistant Enterobacteriaceae

**DOI:** 10.1128/AAC.00236-18

**Published:** 2018-07-27

**Authors:** M. Thwaites, D. Hall, D. Shinabarger, A. W. Serio, K. M. Krause, A. Marra, C. Pillar

**Affiliations:** a Micromyx, Kalamazoo, Michigan, USA; b Achaogen, South San Francisco, California, USA

**Keywords:** plazomicin, bactericidal activity, Enterobacteriaceae, bactericidal, multidrug resistance

## Abstract

The next-generation aminoglycoside plazomicin, in development for infections due to multidrug-resistant (MDR) Enterobacteriaceae, was evaluated alongside comparators for bactericidal activity in minimum bactericidal concentration (MBC) and time-kill (TK) assays against MDR Enterobacteriaceae isolates with characterized aminoglycoside and β-lactam resistance mechanisms. Overall, plazomicin and colistin were the most potent, with plazomicin demonstrating an MBC_50/90_ of 0.5/4 μg/ml and sustained 3-log_10_ kill against MDR Escherichia coli, Klebsiella pneumoniae, and Enterobacter spp.

## TEXT

Carbapenem-resistant Enterobacteriaceae (CRE) and extended-spectrum β-lactamase (ESBL)-producing Enterobacteriaceae (1–3) top the Centers for Disease Control and Prevention's list of major threats (4). Antibiotic resistance is increasing, likely due to the rise of ESBLs and CREs; they are often multidrug resistant (MDR), leaving few therapeutic options and highlighting the need for new agents to treat serious infections caused by these pathogens (5), namely, urinary tract infections, nosocomial pneumonia, bacteremia, and intraabdominal infections. The next-generation aminoglycoside plazomicin has been evaluated in two phase 3 clinical studies in patients with complicated urinary tract infections (cUTI) or acute pyelonephritis (AP) and in patients with bloodstream infections, hospital- and ventilator-associated bacterial pneumonia, or cUTI/AP due to CRE. Aminoglycosides are often used to treat CRE, as these drugs are bactericidal against these strains; however, increasing resistance due to the presence of genes encoding aminoglycoside-modifying enzymes (AMEs) has given clinicians pause (6–8), as these organisms typically carry multiple resistance determinants (9, 10). Plazomicin maintains activity against most aminoglycoside-resistant Enterobacteriaceae as it is not inactivated by plasmid-borne AMEs (11). It is also active *in vitro* against MDR Enterobacteriaceae clinical isolates, including ESBL-producing isolates and CRE. This study examined the bactericidal activities of plazomicin and comparator agents against MDR Enterobacteriaceae in minimum bactericidal concentration (MBC) and time-kill assays.

MDR Enterobacteriaceae isolates were acquired from IHMA (Schaumburg, IL) and were genetically characterized for resistance to aminoglycosides (Achaogen, South San Francisco, CA) and β-lactams (IHMA) (see Table S1 in the supplemental material); these isolates were resistant to currently used antibiotics, including aminoglycosides (amikacin and gentamicin), β-lactams (ceftazidime and meropenem), and a fluoroquinolone (levofloxacin). Escherichia coli ATCC 25922 served as the quality control strain. Plazomicin was provided by Achaogen as a stock solution in sterile distilled water (dH_2_O). The comparators meropenem (USP, Rockville, MD), tigecycline (Waterstone Technology, Carmel, IN), amikacin, colistin, gentamicin, levofloxacin, and ceftazidime (Sigma-Aldrich) were dissolved in accordance with Clinical and Laboratory Standards Institute (CLSI) guidelines (12).

The MIC and MBC values for plazomicin and comparators were determined by broth microdilution in accordance with CLSI guidelines (12–14). For MBC determinations, duplicate 10-μl aliquots from the MIC well and from three wells above the MIC were sampled for CFU enumeration. The MBC was defined as the concentration of drug that resulted in ≥3-log_10_ CFU/ml decrease (99.9% kill) after an overnight incubation. MBC:MIC ratios were determined, and MBC:MIC ratios of ≤4 were considered indicative of bactericidal activity (15).

The time-kill kinetics of plazomicin (at 2-, 4-, 8-, and 16-fold the MIC), amikacin, gentamicin, meropenem, and colistin (at 8-fold the MIC) against 10 isolates (three E. coli, including ATCC 25922, four Klebsiella spp., and three Enterobacter spp.) were determined per CLSI guidelines (14). For isolates with MIC values of >8 μg/ml, a concentration of 64 μg/ml was used. After inoculation and sampling for a baseline viable count, flasks with the appropriate drug concentrations were incubated at 35°C with shaking. The flasks were sampled at specified time points for the determination of viable counts. Bactericidal activity was defined as a 3-log_10_ decrease in CFU/ml relative to the starting inoculum maintained through 24 h.

The MIC_50/90_ and MBC_50/90_ values, as well as the MBC:MIC ratios and percent susceptibilities overall and by species, for plazomicin and comparator agents are shown in Table 1. Against all isolates, plazomicin displayed an MIC_50/90_ of 0.5/2 μg/ml and an MBC_50/90_ of 0.5/4 μg/ml, with an MBC:MIC ratio of ≤4 for 29 of 30 isolates (96.7%). In contrast, amikacin and gentamicin both demonstrated an MIC_50/90_ value of 32/128 μg/ml against these isolates. Amikacin had an MBC_50/90_ of 64/256 μg/ml and an MBC:MIC ratio of ≤4 for 96.7% of isolates; gentamicin had an MBC_50/90_ of 64/>512 μg/ml and an MBC:MIC ratio of ≤4 for 92.6% of isolates. As the majority of the isolates were resistant to these aminoglycosides, the MBCs for gentamicin and amikacin are not clinically relevant, despite the low MBC:MIC ratios.

**TABLE 1:** Summary of the MIC and MBC values (μg/ml) and MBC:MIC ratios of plazomicin and other evaluated agents against Enterobacteriaceae

Organism	Agent^*a*^	MIC				MBC			MBC:MIC ratio		
		*n*	Range (μg/ml)	50%/90% (μg/ml)	%S^*b*^	*n*	Range (μg/ml)	50%/90% (μg/ml)	No. evaluated^*c*^	*n* (%)	
										≤4	>4
Enterobacteriaceae	PLZ	30	0.12 to 8	0.5/2	—	30	0.12 to 8	0.5/4	30	29 (96.7)	1 (3.3)
	AMK	30	1 to 256	32/128	30.0	30	2 to >256	64/256	30	29 (96.7)	1 (3.3)
	GEN	30	0.25 to >512	32/128	33.3	28	0.25 to >512	64/>512	27	25 (92.6)	2 (7.4)
	CAZ	30	0.5 to >512	64/>512	10.0	24	0.5 to >512	128/>512	21	21 (100)	
	MEM	30	0.15 to 128	0.06/64	60.0	30	0.03 to 128	0.12/64	30	30 (100)	
	LVX	30	0.03 to 256	16/64	30.0	30	0.03 to >128	16/64	30	29 (96.7)	1 (3.3)
	TIG	30	0.12 to 8	0.5/4	80.0	30	0.25 to >8	>4/>16	29	7 (24.1)	22 (75.9)
	COL	30	0.06 to >32	0.12/0.25	93.3	28	0.06 to 0.5	0.12/0.5	28	28 (100)	
E. coli	PLZ	10	0.5 to 4	1/4	—	10	0.5 to 8	1/4	10	9 (90.0)	1 (10.0)
	AMK	10	1 to 256	32/128	30.0	10	2 to >256	32/256	10	9 (90.0)	1 (10.0)
	GEN	10	0.25 to >512	64/512	40.0	9	0.25 to >512	64/—	8	8 (100)	
	CAZ	10	0.5 to >512	64/>512	20.0	7	0.5 to >512	64/—	6	6 (100)	
	MEM	10	0.015 to 8	0.03/8	80.0	10	0.03 to 8	0.03/8	10	10 (100)	
	LVX	10	0.03 to 64	16/32	20.0	10	0.03 to >128	32/64	10	9 (90.0)	1 (10.0)
	TIG	10	0.12 to 1	0.25/1	100	10	0.25 to >8	>2/>8	10	3 (30.0)	7 (70.0)
	COL	10	0.12 to 0.25	0.25/0.25	100	10	0.12 to 0.25	0.25/0.25	10	10 (100)	
Klebsiella spp.	PLZ	8	0.25 to 8	0.5/—	—	8	0.25 to 8	0.5/—	8	8 (100)	
	AMK	8	16 to 256	32/—	50.0	8	16 to 256	32/—	8	8 (100)	
	GEN	8	2 to >512	64/—	25.0	7	2 to 256	64/—	7	6 (85.7)	1 (14.3)
	CAZ	8	64 to >512	128/—	0.0	6	2 to >512	64/—	5	5 (100)	
	MEM	8	0.06 to 128	32/—	12.5	8	64 to >512	32/—	8	8 (100)	
	LVX	8	0.5 to 256	16/—	25.0	8	0.06 to 128	16/—	8	8 (100)	
	TIG	8	0.5 to 8	2/—	50.0	8	>4 to >16	>16/—	7	1 (14.3)	6 (85.7)
	COL	8	0.12 to >32	0.12/—	75.0	6	0.12 to 0.5	0.12/—	6	6 (100)	
Enterobacter spp.	PLZ	10	0.25 to 2	0.5/1	—	10	0.25 to 4	0.5/1	10	10 (100)	
	AMK	10	4 to 128	16/64	50.0	10	4 to 256	16/128	10	10 (100)	
	GEN	10	0.5 to 128	8/64	30.0	10	0.5 to >512	8/256	10	9 (90.0)	1 (10.0)
	CAZ	10	4 to 512	64/512	10.0	10	8 to >512	128/512	9	9 (100)	
	MEM	10	0.015 to 32	0.06/4	80.0	10	0.03 to 32	0.06/4	10	10 (100)	
	LVX	10	0.03 to 32	1/32	50.0	10	0.03 to 64	1/32	10	10 (100)	
	TIG	10	0.5 to 8	0.5/4	80.0	10	0.5 to >16	>4/16	10	3 (30.0)	7 (70.0)
	COL	10	0.06 to 0.12	0.12/0.12	100	10	0.06 to 0.12	0.12/0.12	10	10 (100)	
Citrobacter freundii^*d*^	PLZ	2	0.12 to 0.5		—	2	0.12 to 0.5		2	2 (100)	
	AMK	2	32 to 256		0.0	2	64 to >512		2	2 (100)	
	GEN	2	0.5 to 64		50.0	2	0.5 to 64		2	2 (100)	
	CAZ	2	>32 to 512		0.0	1	512		1	1 (100)	
	MEM	2	0.03 to 64		50.0	2	0.03 to 64		2	2 (100)	
	LVX	2	0.5 to 32		50.0	2	0.5 to 32		2	2 (100)	
	TIG	2	0.5 to 2		100	2	>4 to >16		2		2 (100)
	COL	2	0.12 to 0.25		100	2	0.12 to 0.5		2	2 (100)	

a PLZ, plazomicin; AMK, amikacin; GEN, gentamicin; CAZ, ceftazidime; MEM, meropenem; LVX, levofloxacin; TIG, tigecycline; COL, colistin.

b %S, percent susceptibility using CLSI M100-S25 susceptibility breakpoints (FDA breakpoints applied for tigecycline).

c Isolates with MIC/MBC values that were undefined/off scale were not included for analysis of MBC:MIC ratio, i.e., if the MIC value for an isolate fell outside the MIC testing range for an antibiotic.

d Only MIC and MBC ranges are shown for C. freundii (MIC_50/90_ and MBC_50/90_ not applicable).

Ceftazidime and meropenem had MIC_50/90_ values of 64/>512 and 0.06/64 μg/ml, respectively, against the tested isolates. The MBC_50/90_ for ceftazidime was 128/>512 μg/ml; that for meropenem was 0.12/64 μg/ml, with MBC:MIC ratios of ≤4 for 100% of values. Levofloxacin had an MIC_50/90_ of 16/64 μg/ml and an MBC_50/90_ of 16/64 μg/ml, with an MBC:MIC ratio of ≤4 for 96.7% of isolates. Tigecycline and colistin had MIC_50/90_ values of 0.5/4 and 0.12/0.25 μg/ml, respectively, against these Enterobacteriaceae. Tigecycline was generally bacteriostatic by MBC, with an MBC_50/90_ of >4/>16 μg/ml and an MBC:MIC ratio of >4 against 75.9% of isolates. Colistin was bactericidal with an MBC_50/90_ of 0.12/0.5 and an MBC:MIC ratio of ≤4 for all isolates.

**FIG 1:**
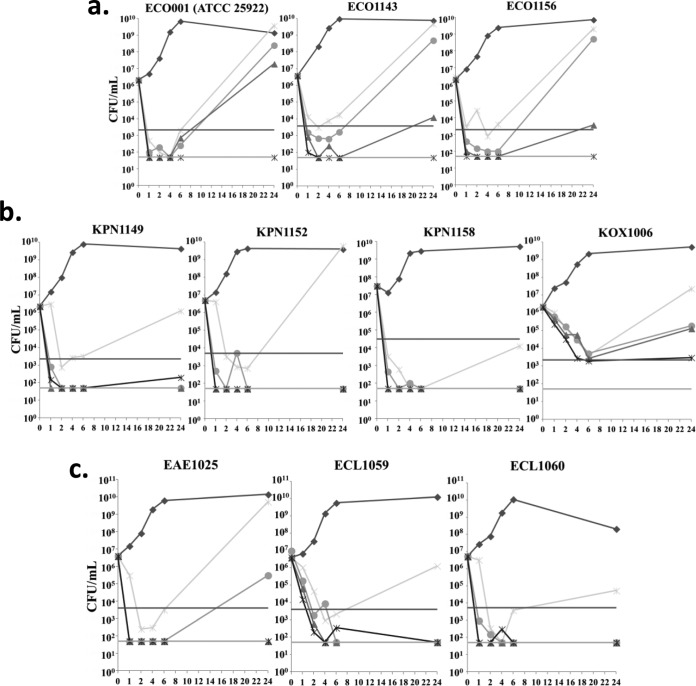
(a) Time-kill kinetics of plazomicin against E. coli. (b) Time-kill kinetics of plazomicin against K. pneumoniae. (c) Time-kill kinetics of plazomicin against Enterobacter spp. Black diamonds, growth control; gray X's, plazomicin at 2× MIC; circles, plazomicin at 4× MIC; triangles, plazomicin at 8× MIC; gray diamonds, plazomicin at 16× MIC. Upper horizontal dashed lines represent the 3-log_10_ CFU decrease from time zero (*t*_0_); lower dotted horizontal lines represent the limits of detection.

Plazomicin and comparators were evaluated by a time-kill assay against a subset of isolates as shown in Table 2; the results for plazomicin are shown in Fig. 1. A summary of the time-kill results for comparators is shown in Fig. S1. At ≥4-fold the MIC against the E. coli isolates (Fig. 1a), plazomicin was rapidly bactericidal for up to 6 h, but there was regrowth at doses <16-fold MIC through 24 h. Against ECO001/ATCC 25922, amikacin (through 24 h) and gentamicin (only to 6 h) showed cidal activity. Colistin showed rapid >3-log_10_ CFU killing against all three strains out to 6 h and against the ECO1143 and ECO156 strains through 24 h. Against the three Klebsiella pneumoniae isolates, plazomicin demonstrated >3-log_10_ CFU killing within 1 h at ≥4-fold the MIC through 24 h (Fig. 1b). Amikacin and gentamicin showed rapid killing against KPN1158 but no activity against KPN1152; amikacin demonstrated killing against KPN1149 as well. Colistin was rapidly cidal only through 6 h against all three K. pneumoniae isolates. Plazomicin demonstrated 2-log_10_ to 3-log_10_ CFU killing by 6 h at all concentrations against the Klebsiella oxytoca isolate, similar to amikacin, which was bactericidal at 6 h; gentamicin was not bactericidal at any time point. Colistin showed bactericidal activity through 6 h against KOX1006 with regrowth at 24 h. Plazomicin demonstrated >3-log_10_ killing against the Enterobacter aerogenes isolate EAE1025 at all concentrations through 6 h without regrowth through 24 h at concentrations ≥8-fold the MIC. Similarly, against the two Enterobacter cloacae isolates ECL1059 and ECL1060, plazomicin was bactericidal at concentrations ≥4-fold the MIC. Amikacin and gentamicin showed 3-log_10_ CFU killing through 6 and 24 h, respectively, against EAE1025 and ECL1060. For colistin, bactericidal activity was observed through 6 h for EAE1025, but cidality was observed for 4 h against ECL1059 and ECL1060.

**TABLE 2:** Activity of plazomicin and comparators against isolates evaluated by time-kill

Organism	Isolate ID^*a*^	AME and β-lactamase molecular summary	MIC (μg/ml)^*b*^				
			PLZ	AMK	GEN	MEM	COL
E. coli	AECO001 (ATCC 25922)		0.5	1	0.25	0.03	0.25
	AECO1143	*aac*(3)-IIa; *aph*(3′)-VIa; *bla*_CTX-M-55_	0.5	256	64	0.015	0.12
	AECO1156	*aac*(3)-IIa; *aac*(6′)-Ib; *bla*_TEM-OSBL_; *bla*_CTX-M-15_	1	64	>512	0.03	0.25
K. pneumoniae	AKPN1149	*aac*(3)-IVa; *aac*(6′)-Ib; *bla*_SHV-OSBL_; *bla*_TEM-OSBL_; *bla*_KPC-2_	0.25	32	64	128	0.12
	AKPN1152	*aac*(3)-IIa; *aac*(6′)-Ib; *aph*(3′)-VIa; *bla*_SHV-OSBL_; *bla*_TEM-OSBL_; bla_CTX-M-14_; *bla*_OXA-48_	0.25	256	128	32	0.12
	AKPN1158	*aac*(6′)-Ib; *bla*_SHV-OSBL_; *bla*_TEM-OSBL_; *bla*_CTX-M-15_; *bla*_KPC-3_	0.5	64	2	16	0.12
K. oxytoca	AKOX1006	*aac*(3)-Ia/d; *aac*(6′)-Ib; *bla*_SHV-5_; *bla*_TEM-OSBL_; *bla*_KPC-2_	1	64	128	16	0.12
E. aerogenes	AEAE1025	*aac*(3)-Ia/d; *aac*(6′)-Ib	0.5	16	8	0.06	0.12
E. cloacae	AECL1059	*aac*(3)-IIa; *aph*(3′)-VIa	0.5	64	128	0.015	0.12
	AECL1060	*aac*(6′)-Ib; *ant*(2″)-Ia; *bla*_SHV-12_; *bla*_ACT-7_	0.5	16	32	32	0.12

a ID, identifier.

b PLZ, plazomicin; AMK, amikacin; GEN, gentamicin; MEM, meropenem; COL, colistin.

Here, plazomicin demonstrated potent bactericidal activity against aminoglycoside- and β-lactam-resistant MDR Enterobacteriaceae isolates. By MBC_50/90_ and by MBC:MIC ratios, plazomicin and colistin were the most active bactericidal agents evaluated; tigecycline had potent activity by MIC but was largely bacteriostatic. In contrast, the high MBC_50/90_ values for the other antibiotics evaluated reflected their decreased activities against this panel of isolates.

Time-kill assays confirmed the potent bactericidal activity of plazomicin, where rapid and sustained 3-log killing at concentrations at or greater than 4-fold the MIC was observed. Plazomicin displays potent *in vitro* activity that further translates to rapid and sustained bacterial killing at lower concentrations than comparator agents in this study. Bactericidal activity at lower concentrations could be an advantage for a new antibacterial agent, as this prevents bacterial regrowth and presumably resistance emergence (16). That this set of organisms is MDR highlights the clinical potential of plazomicin against isolates with challenging resistance phenotypes.

## Supplementary Material

Supplemental file 1

Supplemental file 2
